# *cis*-Bis­(2,2′-bipyrid­yl)dicyanato­cobalt(II)

**DOI:** 10.1107/S1600536808007617

**Published:** 2008-03-29

**Authors:** LI Jia, Ling-Qian Kong, Da-Cheng Li

**Affiliations:** a School of Chemistry and Chemical Engineering, Liaocheng University, Shandong 252059, People’s Republic of China; b Liaocheng Vocational and Technical College, Liaocheng, Shandong 252000, People’s Republic of China; c Dongchang College of Liaocheng University, Liaocheng, Shandong 252000, People’s Republic of China

## Abstract

In the title complex, [Co(NCO)_2_(C_10_H_8_N_2_)_2_], the Co atom is coordinated by four N atoms from two 2,2′-bipyridyl ligands and two N atoms from two cyanate anions in a distorted octa­hedral geometry. The Co atom lies on a twofold rotation axis. The average Co—N bond length is 2.126 (7) Å. Weak inter­molecular C—H⋯O inter­actions lead to the formation of a three-dimensional network.

## Related literature

For the crystal structures of cobalt complexes with analogous ligands, see: Veidis *et al.* (1981 ▶); Tang *et al.* (2004 ▶). For related literature, see: Milani *et al.* (2003 ▶).
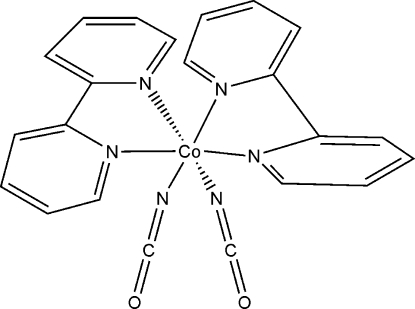


## Experimental

### 

#### Crystal data

[Co(NCO)_2_(C_10_H_8_N_2_)_2_]
*M* *_r_* = 455.34
Orthorhombic, 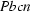

*a* = 14.148 (12) Å
*b* = 9.774 (8) Å
*c* = 15.253 (13) Å
*V* = 2109 (3) Å^3^
*Z* = 4
Mo *K*α radiation
μ = 0.84 mm^−1^
*T* = 298 (2) K
0.30 × 0.25 × 0.06 mm

#### Data collection

Bruker SMART 1000 diffractometer
Absorption correction: multi-scan (*SADABS*; Sheldrick, 1996 ▶) *T*_min_ = 0.786, *T*_max_ = 0.951
10457 measured reflections
1870 independent reflections
1009 reflections with *I* > 2σ(*I*)
*R*_int_ = 0.078

#### Refinement

*R*[*F* ^2^ > 2σ(*F* ^2^)] = 0.039
*wR*(*F* ^2^) = 0.078
*S* = 1.00
1870 reflections
141 parameters
H-atom parameters constrained
Δρ_max_ = 0.25 e Å^−3^
Δρ_min_ = −0.30 e Å^−3^

### 

Data collection: *SMART* (Siemens, 1996 ▶); cell refinement: *SAINT* (Siemens, 1996 ▶); data reduction: *SAINT*; program(s) used to solve structure: *SHELXS97* (Sheldrick, 2008 ▶); program(s) used to refine structure: *SHELXL97* (Sheldrick, 2008 ▶); molecular graphics: *SHELXTL* (Sheldrick, 2008 ▶); software used to prepare material for publication: *SHELXTL*.

## Supplementary Material

Crystal structure: contains datablocks I, global. DOI: 10.1107/S1600536808007617/fi2060sup1.cif

Structure factors: contains datablocks I. DOI: 10.1107/S1600536808007617/fi2060Isup2.hkl

Additional supplementary materials:  crystallographic information; 3D view; checkCIF report

## Figures and Tables

**Table:** 

Co1—N1	2.027 (3)
Co1—N2	2.162 (3)
Co1—N3	2.176 (3)

**Table:** 

N1^i^—Co1—N1	97.0 (2)
N1^i^—Co1—N2	166.18 (11)
N1—Co1—N2	90.76 (12)
N2—Co1—N2^i^	84.07 (14)
N1^i^—Co1—N3	92.83 (12)
N1—Co1—N3	93.59 (11)
N2—Co1—N3	75.22 (11)
N2^i^—Co1—N3	97.44 (10)
N3—Co1—N3^i^	170.29 (13)

Symmetry code: (i) 
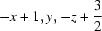
.

**Table 2:** Hydrogen-bond geometry (Å, °)

*D*—H⋯*A*	*D*—H	H⋯*A*	*D*⋯*A*	*D*—H⋯*A*
C10—H10⋯O1^ii^	0.93	2.50	3.236 (5)	137
C5—H5⋯O1^iii^	0.93	2.48	3.198 (5)	134

Symmetry codes: (ii) 
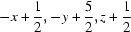
; (iii) 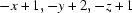.

**Fig. 1.**
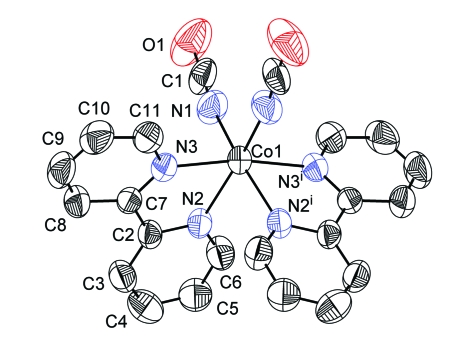
The crystal structure of the title compound showing the atomic numbering and 30% probability displacement ellipsoids. H atoms have been omitted for clarity. The second ligand is generated by i: –x+1, y, –z+3/2.

## supplementary crystallographic information

### Comment

Organometallic Co derivatives are applied as catalysts in polymerization reactions of polar olefins and for the elucidation of the hypothetical mechanism of these polymerization reactions (Milani *et al.*, 2003). In recent years the synthesis of the without bridge bonding mononucleate complexs was used to find out the information to design the multidimensional structure complexes, so the homologic ligands complex [Co(2,2'-bipy)_2_(N_3_)_2_].Cl.2H_2_O was reported(Tang *et al.*, 2004). In this paper, Co(C_10_H_8_N_2_)_2_(NCO)_2_ was synthesized by the reaction of CoCl_2_.6H_2_O, 2,2'-bipyridyl and NaOCN at room temperature and the structure of the resulting complex is presented here (Fig. 1).

The Co atom lies on a special position (Wyckoff position 4c, site symmetry 2). It is formed by coordination of two 2,2'-bipyridyls ligands and two cyanate anions. The coordination gemotry of the central Co atom is distorted octahedral with four N atoms from two 2,2'-bipyridyls and two N atoms from two cyanate anions. The equatorial plane consists of N1, N2, N3 and N3^i^with an average bond length of 2.135 (3) Å. The apical positions are occupied by a cyanate anion and a N atom from a 2,2'-bipyridyl with the bond length 2.027 (3) Å 2.162 (3) Å, respectively. The distances Co—N(2,2'-bipyridyl) in the title complex are significantly longer (2.176 (3) Å and 2.162 (3) Å) than those in the comparable bond length (1.950 (3) Å and 1.954 (3) Å, Tang *et al.*(2004)). The complexes arrange into a three-dimensional network *via* weak intermolecular C—H···O interactions (H···O distances: 2.499 (3) Å and 2.481 (4) Å; C···O distances: 3.417 (5) Å and 3.084 (7) Å)..

### Experimental

CoCl_2_.6H_2_O(0.0476 g 0.2 mmol) was dissolved in 10 ml MeOH, the solution was then added to an aqueous solution of 2,2'-bipyridyl(0.0316 g 0.2 mmol). The reaction mixture was stirred for 10 minutes until the solution color became red. NaOCN(0.0130 g 0.2 mmol) was added to the reaction mixture. The mixture was filtered and red single crystals were obtained by slow evaporation of the mother liquid for three weeks at room temperature. Elemental analysis for C_22_H_16_Co N_6_S_2_ calculated: C 58.03, H 3.54, N 18.45%; found: C 58.23, H 3.23, N 18.30%.

### Refinement

All H atoms were placed geometrically and treated as riding on their parent atoms with C—H 0.93 Å (2,2'-bipyridyl) [*U*_iso_(H) = 1.2*U*_eq_(C)].

### Crystal data

**Table:**

[Co(NCO)_2_(C_10_H_8_N_2_)_2_]	*F*_000_ = 932
*M**_r_* = 455.34	*D*_x_ = 1.434 Mg m^−^^3^
Orthorhombic, *P**b**c**n*	Mo *K*α radiation λ = 0.71073 Å
Hall symbol: -P2n2ab	Cell parameters from 1484 reflections
*a* = 14.148 (12) Å	θ = 2.5–20.8º
*b* = 9.774 (8) Å	µ = 0.85 mm^−^^1^
*c* = 15.253 (13) Å	*T* = 298 (2) K
*V* = 2109 (3) Å^3^	Plate, red
*Z* = 4	0.30 × 0.25 × 0.06 mm

### Data collection

**Table:**

Bruker Smart 1000 diffractometer	1870 independent reflections
Radiation source: fine-focus sealed tube	1009 reflections with *I* > 2σ(*I*)
Monochromator: graphite	*R*_int_ = 0.078
*T* = 298(2) K	θ_max_ = 25.0º
φ & ω scans	θ_min_ = 2.5º
Absorption correction: multi-scan[SADABS; Sheldrick, 1996)	*h* = −16→14
*T*_min_ = 0.786, *T*_max_ = 0.951	*k* = −11→9
10457 measured reflections	*l* = −18→17

### Refinement

**Table:**

Refinement on *F*^2^	Secondary atom site location: difference Fourier map
Least-squares matrix: full	Hydrogen site location: inferred from neighbouring sites
*R*[*F*^2^ > 2σ(*F*^2^)] = 0.039	H-atom parameters constrained
*wR*(*F*^2^) = 0.078	*w* = 1/[σ^2^(*F*_o_^2^) + (0.0386*P*)^2^ + 2.2714*P*] where *P* = (*F*_o_^2^ + 2*F*_c_^2^)/3
*S* = 1.00	(Δ/σ)_max_ = 0.001
1870 reflections	Δρ_max_ = 0.25 e Å^−^^3^
141 parameters	Δρ_min_ = −0.30 e Å^−^^3^
Primary atom site location: structure-invariant direct methods	Extinction correction: none

### Special details

**Table:**

Geometry. All e.s.d.'s (except the e.s.d. in the dihedral angle between two l.s. planes) are estimated using the full covariance matrix. The cell e.s.d.'s are taken into account individually in the estimation of e.s.d.'s in distances, angles and torsion angles; correlations between e.s.d.'s in cell parameters are only used when they are defined by crystal symmetry. An approximate (isotropic) treatment of cell e.s.d.'s is used for estimating e.s.d.'s involving l.s. planes.
Refinement. Refinement of *F*^2^ against ALL reflections. The weighted *R*-factor *wR* and goodness of fit *S* are based on *F*^2^, conventional *R*-factors *R* are based on *F*, with *F* set to zero for negative *F*^2^. The threshold expression of *F*^2^ > σ(*F*^2^) is used only for calculating *R*-factors(gt) *etc*. and is not relevant to the choice of reflections for refinement. *R*-factors based on *F*^2^ are statistically about twice as large as those based on *F*, and *R*- factors based on ALL data will be even larger.

### Fractional atomic coordinates and isotropic or equivalent isotropic displacement parameters (Å^2^)

**Table:**

	*x*	*y*	*z*	*U*_iso_*/*U*_eq_	
Co1	0.5000	1.02936 (6)	0.7500	0.0514 (2)	
N1	0.45099 (19)	1.1668 (3)	0.6615 (2)	0.0780 (10)	
N2	0.42837 (17)	0.8651 (2)	0.68223 (17)	0.0525 (7)	
N3	0.36432 (16)	1.0105 (3)	0.81603 (17)	0.0538 (7)	
O1	0.4129 (2)	1.3351 (3)	0.5593 (2)	0.1331 (13)	
C1	0.4337 (2)	1.2469 (5)	0.6118 (3)	0.0737 (12)	
C2	0.3390 (2)	0.8363 (3)	0.7088 (2)	0.0501 (8)	
C3	0.2898 (2)	0.7277 (4)	0.6735 (2)	0.0655 (10)	
H3	0.2289	0.7081	0.6927	0.079*	
C4	0.3309 (3)	0.6491 (4)	0.6103 (3)	0.0802 (12)	
H4	0.2983	0.5754	0.5863	0.096*	
C5	0.4205 (3)	0.6794 (4)	0.5821 (2)	0.0738 (11)	
H5	0.4496	0.6275	0.5388	0.089*	
C6	0.4657 (2)	0.7879 (4)	0.6196 (2)	0.0669 (10)	
H6	0.5263	0.8090	0.6002	0.080*	
C7	0.3019 (2)	0.9245 (3)	0.7788 (2)	0.0511 (9)	
C8	0.2077 (2)	0.9216 (3)	0.8059 (3)	0.0676 (10)	
H8	0.1645	0.8642	0.7784	0.081*	
C9	0.1797 (3)	1.0036 (4)	0.8731 (3)	0.0808 (13)	
H9	0.1172	1.0020	0.8921	0.097*	
C10	0.2434 (3)	1.0881 (4)	0.9122 (2)	0.0804 (12)	
H10	0.2256	1.1444	0.9585	0.096*	
C11	0.3354 (2)	1.0883 (3)	0.8814 (2)	0.0685 (10)	
H11	0.3790	1.1460	0.9082	0.082*	

### Atomic displacement parameters (Å^2^)

**Table:**

	*U*^11^	*U*^22^	*U*^33^	*U*^12^	*U*^13^	*U*^23^
Co1	0.0391 (3)	0.0536 (4)	0.0615 (4)	0.000	0.0011 (3)	0.000
N1	0.064 (2)	0.078 (2)	0.092 (3)	0.0116 (17)	0.0007 (18)	0.0301 (19)
N2	0.0402 (15)	0.0601 (19)	0.0573 (19)	0.0020 (13)	0.0000 (14)	−0.0038 (14)
N3	0.0457 (15)	0.0555 (19)	0.0603 (19)	0.0050 (14)	0.0027 (14)	−0.0032 (14)
O1	0.115 (2)	0.152 (3)	0.133 (3)	0.055 (2)	0.025 (2)	0.070 (2)
C1	0.052 (2)	0.090 (4)	0.079 (3)	0.020 (2)	0.018 (2)	0.012 (2)
C2	0.0385 (19)	0.056 (2)	0.056 (2)	−0.0010 (17)	−0.0047 (17)	0.0121 (17)
C3	0.051 (2)	0.070 (3)	0.076 (3)	−0.011 (2)	−0.0061 (19)	0.004 (2)
C4	0.087 (3)	0.077 (3)	0.077 (3)	−0.020 (2)	−0.011 (2)	−0.012 (2)
C5	0.074 (3)	0.074 (3)	0.072 (3)	0.001 (2)	0.001 (2)	−0.019 (2)
C6	0.053 (2)	0.079 (3)	0.069 (3)	−0.0057 (19)	−0.0008 (19)	−0.009 (2)
C7	0.0420 (19)	0.047 (2)	0.064 (3)	0.0030 (16)	0.0052 (16)	0.0113 (16)
C8	0.046 (2)	0.071 (3)	0.086 (3)	−0.0028 (18)	0.009 (2)	0.010 (2)
C9	0.053 (2)	0.092 (4)	0.097 (3)	0.012 (2)	0.028 (2)	0.016 (3)
C10	0.079 (3)	0.077 (3)	0.086 (3)	0.014 (2)	0.033 (3)	0.002 (2)
C11	0.062 (2)	0.066 (3)	0.078 (3)	0.0021 (19)	0.013 (2)	−0.009 (2)

### Geometric parameters (Å, °)

**Table:**

Co1—N1^i^	2.027 (3)	C3—H3	0.9300
Co1—N1	2.027 (3)	C4—C5	1.371 (4)
Co1—N2	2.162 (3)	C4—H4	0.9300
Co1—N2^i^	2.162 (3)	C5—C6	1.364 (4)
Co1—N3	2.176 (3)	C5—H5	0.9300
Co1—N3^i^	2.176 (3)	C6—H6	0.9300
N1—C1	1.117 (4)	C7—C8	1.396 (4)
N2—C6	1.327 (4)	C8—C9	1.360 (4)
N2—C2	1.358 (3)	C8—H8	0.9300
N3—C11	1.319 (4)	C9—C10	1.361 (5)
N3—C7	1.345 (3)	C9—H9	0.9300
O1—C1	1.213 (4)	C10—C11	1.384 (4)
C2—C3	1.378 (4)	C10—H10	0.9300
C2—C7	1.469 (4)	C11—H11	0.9300
C3—C4	1.363 (4)		
			
N1^i^—Co1—N1	97.0 (2)	C4—C3—H3	120.2
N1^i^—Co1—N2	166.18 (11)	C2—C3—H3	120.2
N1—Co1—N2	90.76 (12)	C3—C4—C5	119.6 (4)
N1^i^—Co1—N2^i^	90.76 (12)	C3—C4—H4	120.2
N1—Co1—N2^i^	166.18 (11)	C5—C4—H4	120.2
N2—Co1—N2^i^	84.07 (14)	C6—C5—C4	118.1 (4)
N1^i^—Co1—N3	92.83 (12)	C6—C5—H5	121.0
N1—Co1—N3	93.59 (11)	C4—C5—H5	121.0
N2—Co1—N3	75.22 (11)	N2—C6—C5	123.8 (3)
N2^i^—Co1—N3	97.44 (10)	N2—C6—H6	118.1
N1^i^—Co1—N3^i^	93.59 (11)	C5—C6—H6	118.1
N1—Co1—N3^i^	92.83 (12)	N3—C7—C8	121.0 (3)
N2—Co1—N3^i^	97.44 (10)	N3—C7—C2	116.1 (3)
N2^i^—Co1—N3^i^	75.22 (11)	C8—C7—C2	123.0 (3)
N3—Co1—N3^i^	170.29 (13)	C9—C8—C7	119.3 (3)
C1—N1—Co1	172.6 (3)	C9—C8—H8	120.3
C6—N2—C2	117.9 (3)	C7—C8—H8	120.3
C6—N2—Co1	125.4 (2)	C8—C9—C10	119.7 (3)
C2—N2—Co1	116.6 (2)	C8—C9—H9	120.2
C11—N3—C7	118.4 (3)	C10—C9—H9	120.2
C11—N3—Co1	125.1 (2)	C9—C10—C11	118.4 (4)
C7—N3—Co1	115.9 (2)	C9—C10—H10	120.8
N1—C1—O1	178.3 (5)	C11—C10—H10	120.8
N2—C2—C3	120.9 (3)	N3—C11—C10	123.2 (3)
N2—C2—C7	115.3 (3)	N3—C11—H11	118.4
C3—C2—C7	123.7 (3)	C10—C11—H11	118.4
C4—C3—C2	119.6 (3)		

Symmetry codes: (i) −*x*+1, *y*, −*z*+3/2.

### Hydrogen-bond geometry (Å, °)

**Table:**

*D*—H···*A*	*D*—H	H···*A*	*D*···*A*	*D*—H···*A*
C10—H10···O1^ii^	0.93	2.50	3.236 (5)	137
C5—H5···O1^iii^	0.93	2.48	3.198 (5)	134

Symmetry codes: (ii) −*x*+1/2, −*y*+5/2, *z*+1/2; (iii) −*x*+1, −*y*+2, −*z*+1.
